# Cytochemical and ultrastructural aspects of aquatic carnivorous plant turions

**DOI:** 10.1007/s00709-014-0646-8

**Published:** 2014-04-26

**Authors:** Bartosz J. Płachno, Lubomír Adamec, Małgorzata Kozieradzka-Kiszkurno, Piotr Świątek, Iwona Kamińska

**Affiliations:** 1 Department of Plant Cytology and Embryology, Jagiellonian University Kraków, 9 Gronostajowa St, 30-387 Cracow, Poland; 2 Institute of Botany, Section of Plant Ecology, Academy of Sciences of Czech Republic, Dukelská 135, 379 82 Třeboň, Czech Republic; 3 Department of Plant Cytology and Embryology, University of Gdańsk, Wita Stwosza 59 St, 80-308 Gdańsk, Poland; 4 Department of Animal Histology and Embryology, University of Silesia, Bankowa 9, 40-007 Katowice, Poland

**Keywords:** Aquatic carnivorous plants, Winter buds, Storage functions, Protein storage vacuoles, *Aldrovanda*, *Utricularia*

## Abstract

Turions, which are modified shoot apices, are vegetative, dormant overwintering organs produced by perennial aquatic plants. In this study, the turion cytochemistry and ultrastructure of *Aldrovanda vesiculosa*, *Utricularia vulgaris* and *U. stygia* were compared with particular emphasis placed on storage substances. These three aquatic, rootless carnivorous plant species were studied at the end of their winter dormancy. At this stage, the turions of all species had starch as their main storage material. In contrast with *A. vesiculosa*, *Utricularia* turions were rich in protein storage vacuoles, and proteins were also accumulated as crystalline inclusions in the nuclei. All examined species accumulated lipid droplets in cells of epidermal glands.

## Introduction

Turions (winter buds) are vegetative, dormant organs produced by perennial aquatic plants in response to unfavourable ecological conditions (Sculthorpe 1967; Bartley and Spence 1987). They are formed by the extreme condensation of short, modified leaves in the shoot apex, and these tough, sturdy organs form at the end of the growing season. As overwintering organs, turions are partly frost resistant, while their fragile mother shoots are not (Winston and Gorham 1979; Adamec 1999a; Adamec and Kučerová 2013). Turions of rootless, aquatic carnivorous plants of the genera *Aldrovanda* and *Utricularia* usually overwinter and break their innate dormancy at the bottom of an aquatic habitat in darkness, under hypoxic or anoxic conditions. Nevertheless, turions of aquatic plants can also overwinter above the surface on wet substrate and can possess a certain degree of frost resistance which is induced by the hardening effect of weak frosts (Adamec 1999a, b; Adamec and Kučerová 2013). Moreover, turions of several species can even be drought resistant (Maier 1973a, b; Adamec 2008a). Two distinct ecophysiological strategies of autumnal turion sinking and spring rising can be distinguished among aquatic carnivorous plants (Adamec 1999a, 2003, 2008b). In *Aldrovanda vesiculosa*, an active mechanism of turion sinking and rising has developed. In autumn, ripe turions break from the dying mother shoots at the surface and, after a few days, they sink to the bottom. Their high density is not only caused by their high starch content but also by the infilling of their voluminous gas spaces with water. In the spring, at the point of breaking their imposed dormancy, they respond to water warming. Excess water is expelled from the gas spaces by gas evolution, which results from an increasing dark respiration rate, and within a few days, they rise to the surface where they germinate and sprout (Adamec 2003, 2008b). The majority of temperate *Utricularia* species form turions which are less dense than water and are firmly connected to the decaying mother shoots, which drag the turions to the bottom. By early spring, the turions separate and float to the surface. Some of these turions, however, can separate in autumn, and these overwinter at the water surface and are included in the ice. Generally, turion overwintering in the field is considered to be the critical phase of the plant’s seasonal cycle (Adamec 1999a, b).

Turions are also storage organs and, in autumn, they accumulate starch (9–70 % dry weight, DW) and free sugars (in total 7–14 % DW; Winston and Gorham 1979; Ley et al. 1997; Adamec 1999a; 2003; Weber and Noodén 2005). During the overwintering period, starch content gradually decreases and the dark respiration rate of turions of aquatic carnivorous species is several times lower than the dark respiration rates of shoots (leaves) of the same or other species at a standard temperature (Adamec 2003, 2008b, 2011). The turions of aquatic plants also act as storage organs for mineral nutrients (N and P), although this storage function is presumably less important than that for carbohydrates (Adamec 2010).

In contrast to turion morphology, relatively little is known about turion anatomy (cf. Sculthorpe 1967, Vintejoux 1982, 1984; Adamec 2008b). In *Aldrovanda* turions, the narrow leaflets consist of one-cell-layered epidermal walls which surround long, voluminous gas lacunae (diameter ca. 100–300 μm) filled either with water or gas (Adamec 2003); the proportion of these leaf lacunae on the leaf cross-section can reach ca. 40–60 %. Some leaf lacunae were filled with a slimy reticulum stainable with Alcian Blue. It may be hypothesised that the function of this reticulum relates either to stabilisation of the water volume over winter or gas evolution in the spring.

The aim of the study was to describe in detail the cytological ultrastructure of the turions of three species of aquatic carnivorous plants which differ in their ecophysiological traits. An emphasis is put on storage substances. Turions of *A. vesiculosa* possess the mechanism of actively sinking and rising, while turions of *U. vulgaris* and *U. stygia* are dragged by their mother shoots to the bottom. Moreover, turions of *U. vulgaris*, as a strictly aquatic plant, usually overwinter in deeper water, while turions of the latter amphibious or even semi-terrestrial species can also overwinter on wet soil (Adamec 1999a, b, 2007; Adamec and Kučerová 2013). The question of whether these ecophysiological differences among the turions are also reflected in their cytochemistry is also therefore discussed.

## Materials and methods

### Plant material

Unripe turions of *A. vesiculosa* L. (Droseraceae) were collected from the field in the Třeboň Basin Biosphere Reserve and Protected Landscape Area, S Bohemia, Czech Republic (approx. 49° N, 14° 45′ E) in early October. They were allowed to fully ripen outdoors in a plastic container used for growing *Utricularia* species (Adamec 2003). Ripe turions of *Utricularia vulgaris* L. (origin in S Moravia, Czech Rep.) and *U. stygia* Thor (Thor 1988; syn. *U. ochroleuca* Hartm. s*.*l*.*, Lentibulariaceae; origin in Třeboň Basin) were collected from a nearly natural culture in a 2.5 m^2^ outdoor plastic container at the Institute of Botany at Třeboň in mid-November. For the cultivation conditions see, Sirová et al. (2003). Ripe turions of all three species were washed with tap water and stored in filtered cultivation medium (from the outdoor culture of *U. vulgaris* and *U. stygia*) in a dark refrigerator at 2 to 3 °C during winter. Turions were fixed in March 2013, and it was verified that all turions had been innately dormant in mid-November (Adamec 2011). For the general turion morphology, see Adamec (2008b). All items originating from the collection of aquatic and wetland plants of the Institute of Botany at Trebon, Czech Republic, are listed in the collection on “http://www.wetcol.butbn.cas.cz/species”, but voucher numbers are not established.

### Anatomy and cytochemistry

About five turions of each species were fixed in 2.5 % glutaraldehyde and 2.5 % paraformaldehyde in 0.05 M cacodylate buffer (pH 7.0) at room temperature for 4 h. The plant material was rinsed with the same buffer, dehydrated with acetone and embedded in Spurr’s resin. Cross and longitudinal sections 1 μm thick were cut with glass knives and mounted on glass slides. For light microscopy, the sections were stained with 0.05 % toluidine blue O on a hot plate at 60 °C for 1 min. Cytochemical tests included periodic acid–Schiff (PAS) reaction for insoluble polysaccharides (Jensen 1962) and aniline blue black (Jensen 1962) for proteins and Sudan Black B for lipids (Bronner 1975). Sections were examined and photographed with a Nikon Eclipse 800 or an Olympus BX60 microscope.

### Ultrastructure

The procedure for preparing samples for TEM was described by Płachno and Świątek (2008, 2010). Briefly, turions were fixed in 2.5 % formaldehyde and 2.5 % glutaraldehyde in a 0.05 M cacodylate buffer (pH 7.0) for 2 days. The material was post-fixed in 1 % OsO_4_ in a cacodylate buffer at ~4 °C for 24 h, rinsed with the same buffer, treated with 1 % uranyl acetate in distilled water for 1 h, dehydrated with acetone and embedded in Spurr’s resin. Ultrathin sections were cut on a Leica ultracut UCT ultramicrotome. After contrasting with uranyl acetate and lead citrate, the sections were examined using a Hitachi H500 electron microscope at 75 kV.

## Results

**Fig. 1:**
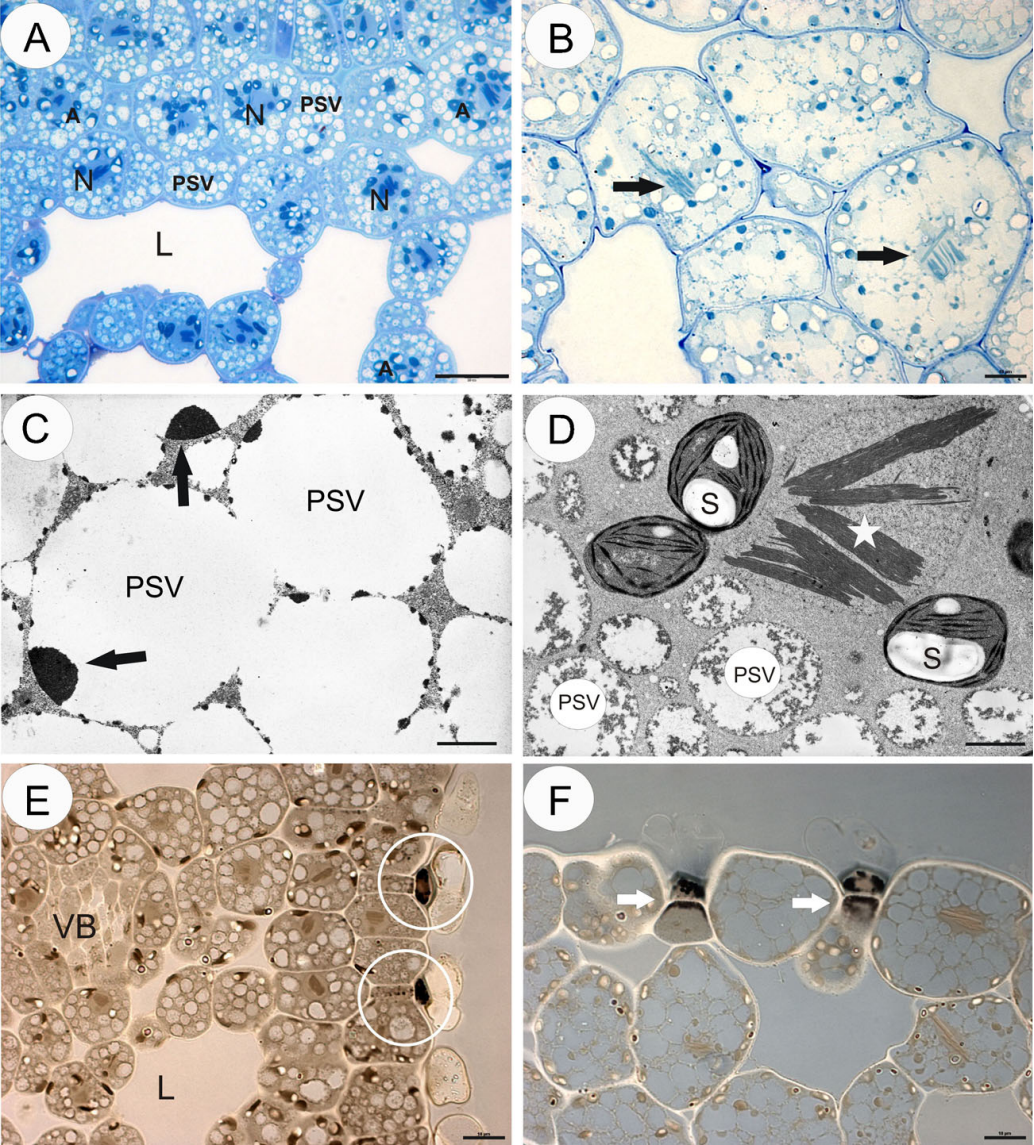
Cytochemistry and ultrastructure of *Utricularia* turions. **a** Semi-thin section through *U. vulgaris* turion; the cytoplasm is filled with numerous light-translucent protein bodies (PSVs). The nuclei (*N*) are surrounded by amyloplasts (*A*), lacunae (*L*), *bar* = 10 μm. **b** *U. stygia* turion tissue stained with aniline blue black showing protein distribution; the protein storage vacuoles (*PSVs*) contained electron-dense remnants of storage proteins, note protein para-crystalline inclusions in the nuclei (*arrows*), *bar* = 20 μm. **c** Ultrastructure of *U. stygia* turion cells; the protein storage vacuoles (*PSVs*) contained electron-dense remnants of storage proteins (*arrows*), *bar* = 2.7 μm. **d** Ultrastructure of *U. vulgaris* turion cells; the protein storage vacuoles (*PSVs*), plastid with starch grains (*S*), para-crystalline inclusions in the nuclei (*star*), *bar* = 0.9 μm. **e** *U. vulgaris* section stained with Sudan Black B showing lipid distribution—lipid droplets occurred in the epidermal glands (*circle*), lacunae (*L*), vascular bundle (*VB*), *bar* = 10 μm. **f** *U. stygia* section stained with Sudan Black B showing lipid distribution—black lipid droplets occurred in basal and middle cells of the epidermal glands (*arrows*), *bar* = 10 μm

The turions consisted of rosettes of short, concentric leaflets in the case of *Aldrovanda* or short, leaf-like phylloclades in the case of *Utricularia*. In both *Utricularia* species, the cytoplasm of turion cells was fully filled by the protein storage vacuoles (Figs. 1a, b) at varying degrees of degradation. However, there was a difference between the species. In *U. stygia*, the protein storage vacuoles were partially degraded and contained electron-dense remnants of storage proteins (Fig. 1c). In *U. vulgaris*, the protein storage vacuoles contained a fibrous material, especially present in the external layer, but the central part of the protein storage vacuoles was electron translucent (Fig. 1d). Both species contained para-crystalline inclusions in the nuclei, which were of a proteinaceous character (Figs. 1b, d). In both species, numerous lipid bodies occurred in basal and middle cells of the epidermal glands (Figs. 1e, f). Amyloplasts surrounded the nuclei, which contained well-developed stacks of thylakoids (Figs. 1a, d).

**Fig. 2:**
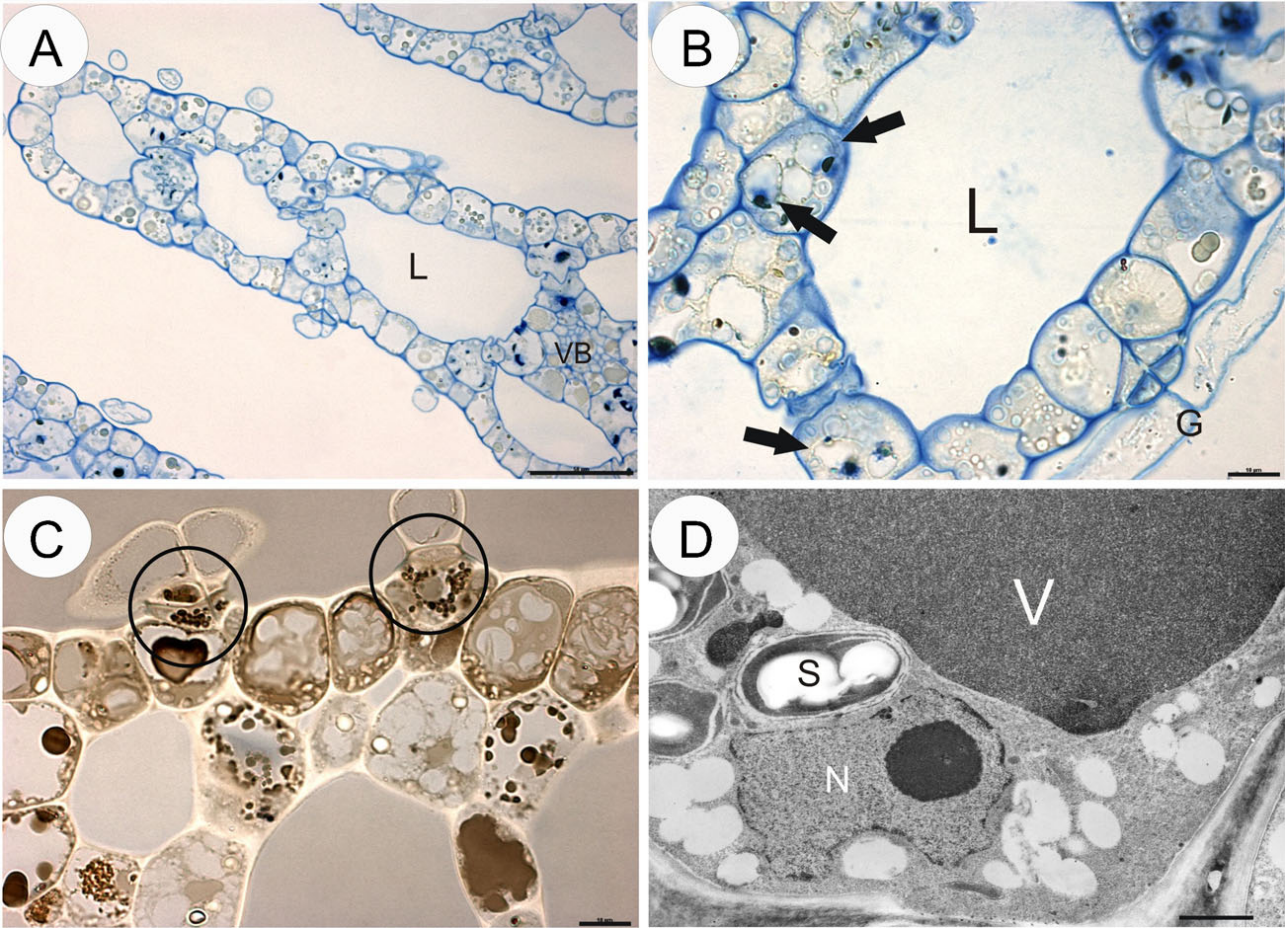
Cytochemistry and ultrastructure of *Aldrovanda vesiculosa* turions. **a** Semi-thin section stained with aniline blue black showing protein distribution. Protein storage vacuoles occurred in mesophyll cells near vascular bundle (*VB*), lacunae (*L*), *bar* = 50 μm. **b** Semi-thin section stained with aniline blue black showing protein storage vacuoles (*arrows*), lacunae (*L*), epidermal gland (*G*), *bar* = 10 μm**. c** Section stained with Sudan Black B showing lipid distribution, lipid droplets spread in the epidermal glands (*circle*), *bar* = 10 μm. **d** Ultrastructure of turion cell; plastid with starch grains (*S*), nuclei (*N*), vacuole (*v*) with tannin-like material, *bar* = 0.8 μm

In *Aldrovanda*, protein storage vacuoles were not frequent and occurred in mesophyll cells, especially near vascular bundles (Fig. 2a). The protein storage vacuoles were partially degraded and contained electron-dense remnants of storage proteins (Fig. 2b). Numerous lipid bodies occurred in basal cells of the epidermal glands (Fig. 2c). Amyloplasts occurred near the nucleus and contained a poorly developed membrane system and a few starch grains (Fig. 2d). The nuclei of mesophyll cells were lobed. The cytoplasm contained small numbers of mitochondria, profiles of rough ER and Golgi bodies. All cells were rich in tannin-like material in large vacuoles (Fig. 2d); however, smaller vacuoles without this material also occurred.

## Discussion

Aquatic plants differ in their development from terrestrial plants in their morphology and physiology. Unlike terrestrial plants, their dormant phase is frequently turions rather than seeds, and turions are formed without sexual reproduction (Wang and Messing 2012). The content and chemical composition of plant reserve materials can vary markedly, but they are accumulated in specific organelles that may be present in embryonic or reserve tissues, such as the endosperm and perisperm (Bewley and Black 1983). Plants store proteins in embryo and vegetative cells to provide carbon, nitrogen and sulphur containing resources for later growth and development. The proteins are typically stored in protein storage vacuoles, which serve as intermediate storage compartments for nitrogen and carbon reserves and other minerals. All of these are needed for early seedling growth (Herman and Larkins 1999; Wang et al. 2007).

The protein content of the cytoplasm in the turion cells of *U. vulgaris* is higher than that in the *U. stygia* and *Aldrovanda* (cf. Figs. 1 and 2)*.* The turion cells in both *Utricularia* species have abundant storage proteins deposited in the form of the protein storage vacuoles, and these were evenly distributed throughout the turions’ cells. In *U. stygia* turions, these organelles included electron-dense remnants of storage proteins. The protein storage vacuoles, such as those in *U. stygia*, are similar to those found in the heart and late heart stages of the embryo of *Jovibarba sobolifera* (Kozieradzka-Kiszkurno et al. 2012). While in the *U. vulgaris*, the protein storage vacuoles contained a fibrous material, in *Aldrovanda*, deposits of proteins were not numerous and most of them were localised in the parenchyma cells.

The presence of lipids in the turions was confirmed by Sudan Black B and osmium tetroxide tests; the lipids were stored (deposited) in the form of droplets. Our observations clearly confirmed that in both *Utricularia* spp. and *Aldrovanda*, lipid droplets occurred almost exclusively in the basal and middle cells of the epidermal glands. Thus, lipids are not the main stored energetic material in carnivorous plant turions. Recent studies have revealed that lipid droplets, previously considered static storage depots of cellular neutral lipids, are organelles actively engaged in lipid metabolism, lipid storage, membrane traffic, protein degradation and cellular signalling (Murphy, 1990). The compartmentation of neutral lipids in plants is mostly associated with seed tissues, where triacylglycerols stored within lipid droplets serve as an essential physiological energy and carbon reserve during germination and post-germination growth (Chapman et al. 2012). The lipids are synthesised on membranous compartments of the cytoplasm and accumulated in lipid bodies, where there is a predominance of triacylglycerides which provide energy and structural membrane blocks during the initial stages of germination and embryo growth (Murphy 1990).

Turions, as vegetative dormant organs, possess a low number of organelles. In the turion cells from all three species, the nucleus was the most prominent organelle within the cell. Para-crystalline inclusions were only found in the *Utricularia* species. Similar inclusions were described previously in several members of the Lentibulariaceae, e.g. in *Pinguicula* (Thomas and Gouranton 1979) and in various tissues of *Utricularia*, including turions of *U. australis* (Genevès and Vintejoux 1967; Vintejoux 1984). Para-crystalline inclusions occur in various plant species, both in ferns and flowering plants, and play a possible role in reserve or storage forms of proteins (Kim 2011).

Although proteins are the dominant storage products, some amyloplasts were present in the turions. All turion amyloplasts investigated here showed a positive reaction for protein (aniline blue black) and were located close to the nucleus. The presence of large proteinaceous plastids was also observed in the embryos of *Pisum sativum* (Marinos 1970), *Stellaria media* (Newcomb and Fowke 1974) and many species of Crassulaceae (Kozieradzka-Kiszkurno and Płachno 2013). Amyloplasts in seeds, roots and stems, which lack chlorophyll and internal membranes, are the main organelles responsible for the synthesis and storage of starch granules in most plants (Wang and Messing 2012). The plastids in turions, where starch synthesis takes place, still retain abundant stacks of thylakoids (Fig. 1d). The sinking and floating (density changes) of aquatic plant turions are also connected with storage and utilisation of starch. Therefore, it should not be surprising that starch was recorded in the turion tissue of various aquatic plants, e.g. *Aldrovanda* (Adamec 2003), *U. vulgaris* (Winston and Gorham 1979), *Myriophyllum verticillatum* (Weber and Noodén 2005), *Spirodela* (Ley et al. 1997; Wang and Messing 2012) and *Potamogeton distinctus* (Harada and Ishizawa 2003). It is worth mentioning that the majority of the *Utricularia* species utilise starch as a storage material in their embryos (Płachno and Świątek 2010). Some characters of turion structure are similar to the gemmae of another carnivorous genus *Drosera*; e.g. both turions and gemmae have storage tissue with starch as a storage material (Bobák et al. 1998) and also both play a role in plant propagation. Temperate *Pinguicula* species (genus related to *Utricularia*) form hibernaculum (i.e. terrestrial winter bud) for overwintering, which resembles *Utricularia* turions. However, the ultrastructure of the *Pinguicula* hibernacula has not yet been examined.

In conclusion, unlike *Aldrovanda*, *Utricularia* turions are rich in protein storage vacuoles and proteins were also accumulated as crystalline inclusions in the nuclei. Despite the existence of ecophysiological differences among *U. vulgaris* and *U. stygia*, no significant differences in their turion cytochemistry were recorded.
